# An Angiotensinogen Gene Polymorphism (rs5050) Is Associated with the Risk of Coronary Artery Aneurysm in Southern Chinese Children with Kawasaki Disease

**DOI:** 10.1155/2019/2849695

**Published:** 2019-01-03

**Authors:** Yunfeng Liu, Lanyan Fu, Lei Pi, Di Che, Yufen Xu, Hao Zheng, Haifeng Long, Lanlan Zeng, Ping Huang, Li Zhang, Tao Yu, Xiaoqiong Gu

**Affiliations:** ^1^Clinical Laboratory, Guangzhou Women and Children's Medical Center, Guangzhou Medical University, Guangzhou, China; ^2^Clinical Biological Resource Bank, Guangzhou Institute of Pediatrics, Guangzhou Women and Children's Medical Center, Guangzhou Medical University, Guangzhou, China; ^3^Department of Cardiology, Guangzhou Women and Children's Medical Center, Guangzhou Medical University, Guangzhou, China; ^4^CapitalBio Genomics Co., Ltd, Dongguan 523808, China

## Abstract

**Background:**

Kawasaki disease (KD) is an acute vasculitis disease that commonly causes acquired heart disease in children. Coronary artery aneurysm (CAA) is a major complication of KD. However, the pathogenesis of KD remains unclear. The results of a genome-wide association study (GWAS) showed that two functional single-nucleotide polymorphisms (SNPs; rs699A>G and rs5050T>G) in the angiotensinogen (AGT) gene were related to cardiovascular disease susceptibility. The purpose of our study was to estimate the relationship between the two GWAS-identified AGT gene polymorphisms and the risk of CAA in Southern Chinese children with KD.

**Methods:**

We genotyped the two AGT gene polymorphisms (rs699A>G and rs5050T>G) in 760 KD cases and 972 healthy controls. We used the odds ratios (ORs) and 95% confidence intervals (CIs) to estimate the degree of the associations.

**Results:**

These two AGT gene polymorphisms were not associated with a risk of KD relative to the controls, but after adjusting for sex and age, the carriers of the rs5050G allele with TG/GG vs TT had an adjusted OR = 1.56, 95% CI = 1.01-2.41, and *P* = 0.044 relative to the carriers of the rs5050TT genotype. The susceptibility to CAA was more predominant in KD patients younger than 12 months old.

**Conclusions:**

Our results indicate that the AGT gene polymorphism rs5050T>G may increase the risk of CAA in children with KD, especially those who are younger than 12 months. These results need to be verified by a validation study with a larger sample size.

## 1. Introduction

Kawasaki disease (KD) is an acute, autoimmune-like, self-limited vasculitis disease with an unknown etiology [1, 2]. This disease mainly involves the middle and small arteries, especially the coronary artery, and some patients develop coronary artery aneurysms (CAAs) and coronary artery stenosis or thrombosis [3]. KD mainly occurs between 6 months and 5 years of age, and a major complication of KD is CAA [4, 5]. Recently, the disease has become among the most common causes of acquired heart disease in children [6]. The results of a genome-wide association study (GWAS) showed that single-nucleotide polymorphisms (SNPs) in the genes nebulette (NEBL) (rs16921209), inositol 1,4,5-trisphosphate 3-kinase C (ITPKS) (19q23), transforming growth factor-*β* (TGF-*β*) (19q13.1), and potassium calcium-activated channel subfamily N member 2 (KCNN2) (5q22.3) were closely related to coronary artery lesions in KD [7–10]. The results of another study showed that NEBL genetic polymorphisms may be related to KD-associated CAA complications and that NEBL plays an important role in the development of CAAs during KD progression [7]. Researchers have also found that the SNP rs28493229 in ITPKC is associated with KD and coronary artery complications in Koreans, and a significant association was found between rs7251246 in ITPKC and CAL formation in a Taiwanese population [11, 12]. Another candidate SNP, i.e., TGF-*β* (rs6550004), was associated with the development of KD, and TGF-*β* (rs1495592) was associated with coronary artery lesions (CALs) in children with KD [13, 14]. The AGT gene is an important vascular lesion-related factor [15–17]. The AGT gene is located in the 1q42-43 region of the long arm of chromosome 1 and has a total length of 13 kb; the gene coding region is composed of 5 exons and 4 introns. Numerous SNPs have been found in the exons and introns. The biological effects of these polymorphisms on the human body are very complex. Among these SNPs, two missense mutations are the most concerning. One mutation is rs699 (M235T), while the other mutation is rs5050 (A-20C). The AGT gene encodes angiotensinogen (AGT), which is a rate-limiting substrate in the renin-angiotensin system (RAS) [18]. AGT catalyzes angiotensin I (Ang I) through renin, and then Ang I is catalyzed to angiotensin II (Ang II) by Ang-converting enzyme (ACE). Ang II is the main active substance in the RAS [19]. Ang II plays a key role in the acute and chronic regulation of arterial blood pressure [20, 21]. KD is characterized as a vascular injury disease that is similar to continuous hypertension. Since an AGT gene mutation is associated with vascular injury, there is a possible correlation between the AGT gene and KD. However, sufficient evidence explaining the possible relationship between the AGT gene single-nucleotide polymorphism and KD is lacking [22, 23]. Therefore, we selected two functional polymorphisms of the AGT gene (rs699A>G and rs5050T>G) based on a thorough evaluation of polymorphisms associated with vasculopathy [18, 20–22] in this hospital-based study. We estimated the relationship between these two polymorphisms and the susceptibility to CAA in Southern Chinese children with KD using 760 cases and 972 controls. Based on these findings, we should pay special attention to the possibility of CAA in children with KD based on AGT gene mutations. The occurrence of CAA may be prevented by applying effective interventions early, thereby reducing the risk of death caused by CAA in children with KD.

## 2. Materials and Methods

### 2.1. Study Population

This study included 760 patients with KD who were mainly recruited at the Cardiology Department of the Guangzhou Women and Children's Medical Center between 2015 and 2016. All KD cases were newly diagnosed and confirmed by the American Heart Association's Kawasaki disease diagnostic criteria in 2004 [24], and the patients did not have a previous history of other cardiovascular diseases. No restriction was applied regarding sex or disease stage at the time of KD case recruitment. In total, 972 age-, ethnicity-, and sex-matched healthy controls were also recruited at the Guangzhou Women and Children's Medical Center. Each subject donated 2 ml of blood for genomic DNA extraction. In addition, the CAA definition was based on that of the Japanese Kawasaki Disease Research Committee as follows: according to the internal diameters of the coronary vessels, the patients with CALs were divided into groups with dilatations or small coronary artery aneurysms (SCAA) (<5.0 mm), mid-sized coronary artery aneurysms (MCAA) (5.0-8.0 mm), and giant coronary artery aneurysms (GCAA) (>8.0 mm). This study was performed with the approval of the Institutional Committee of Guangzhou Women and Children's Medical Center (2014073009). All participants provided written informed consent.

### 2.2. SNP Selection and Genotyping

We selected two polymorphisms in the AGT gene, i.e., rs699 (M235T in exon 2) and rs5050 (A-20C in the promoter region), for detection. The selection of the two functional polymorphisms was based on a thorough evaluation of polymorphisms associated with vasculopathy [22, 25–27]. Genomic DNA was extracted from all KD cases and controls blood using the TIANamp Blood DNA Kit (Qiagen, Dusseldorf, Germany). We stored the samples at −80°C until batch genotyping. The DNA samples were genotyped through PCR using multiple gene-specific primer pairs for AGT (rs699: forward CTTACCTTGGAAGTGGACGTAGG/reverse TCTGGACTTCACAGAACTGGATG; rs5050: forward TCTGCTGTAGTACCCAGAACAAC/reverse CAAGTGATGTAACCCTCCTCTCC). The volume of the PCR mixture was 10 *μ*l (2 × multiplex PCR mix + PCR primer pool and template DNA). The following steps were used for the PCR: 95°C for 3 min; 15 cycles of 95°C for 20 s, 58°C for 90 s, and 72°C for 30 s; and 72°C for 1 min using a GeneAmp PCR System 9700 (Thermo Fisher Scientific). The PCR products were subjected to massive parallel sequencing using an Ion Proton system (Life Technologies, CA, USA).

### 2.3. Statistical Analysis

The *χ*^2^ test was used to compare the differences in the genotypes and frequencies of the alleles and the differences in the demographic variables and other covariates between the cases and controls. We employed the goodness-of-fit *χ*^2^ test to calculate the Hardy-Weinberg equilibrium (HWE) of the genotype distributions in both groups. We calculated the crude and adjusted odds ratios (ORs) and 95% confidence intervals (CIs) by univariate and multivariate logistic regression models to evaluate the associations between the genotypes and KD with and without the CAA adjustment stratified by age and sex. A haplotype analysis was not performed because the selected SNPs appeared to be in the same block. All analyses were performed using SAS software (version 9.1; SAS Institute, Cary, NC), and all statistical tests were two sided. *P* values less than 0.05 were considered statistically significant.

## 3. Results

### 3.1. Population Characteristics

As shown in Table 1, in total, 760 KD cases and 972 age-, sex-, and ethnicity-matched controls were included in our study. No significant differences were observed in age (28.04 ± 23.96 vs 26.25 ± 20.84, *P* = 0.133) or sex (*P* = 0.546) between the KD cases and healthy controls. Among the KD cases, 104 (13.70%) patients were diagnosed with CAA, whereas 656 (86.30%) patients were not diagnosed with CAA.

### 3.2. Associations between AGT Gene Polymorphisms and KD Susceptibility

As shown in Table 2, the genotype frequency distributions of the two SNPs were in accordance with the HWE in the control subjects (*P* = 0.86 for rs699A>G and *P* = 0.18 for rs5050T>G). No significant difference in the genotype distributions of the two investigated SNPs was observed between the KD cases and controls. After adjusting for age and sex, no association was found in the two SNPs between the cases and controls.

### 3.3. Susceptibility to CAA in KD Patients with Two AGT Gene Polymorphisms

As shown in Table 3, among the two investigated SNPs, a significant difference in the genotype distributions of the rs5050T>G polymorphism was observed between the KD cases with CAA and KD cases without CAA. After adjusting for age and sex, the carriers of the rs5050G allele exhibited TG/GG vs TT with an adjusted OR = 1.56, 95% CI = 1.01-2.41, and *P* = 0.043 relative to the carriers of the rs5050TT genotype, suggesting that this SNP had an enhancing effect on the complication of CAA in KD. However, no significant difference in the genotype distributions of the rs699A>G polymorphism was observed between the KD cases with CAA and those without CAA. After adjusting for age and sex, the carriers of the rs699G allele had AA/AG vs GG with an adjusted OR = 2.50, 95% CI = 0.32-19.40, and *P* = 0.381 relative to the carriers of the rs699GG genotype.

### 3.4. Stratification Analysis

As shown in Table 4, we explored the association between the rs5050T>G polymorphism and CAA susceptibility in KD cases in a stratification analysis by age and sex. Compared with the effect of the rs5050 TT genotype, the increased effect of TT/TG genotypes was more predominant among the children aged < 12 months (adjusted OR = 2.03, 95% CI = 1.03-4.03, *P* = 0.042). In terms of coronary artery lesions, we observed a significantly increased risk of MCAA (adjusted OR = 2.12, 95% CI = 1.05-4.30, *P* = 0.037) based on genotype. The combined results indicated that the genotype increased the risk of CAA in children aged < 12 months.

## 4. Discussion

Kawasaki disease can occur in all races worldwide, but its incidence in the Asian race is high. Even in the United States, the prevalence among Asian infants is 3 times higher than that among blacks and 6 times higher than that among whites [28]. Clearly, genetic factors are highly important elements affecting the incidence of KD. The genetic background of KD and its complications are complex, and susceptibility can be related to multiple genes. Studies investigating KD susceptibility genes have been divided into the following two categories: one involves genes implicated in inflammatory reactions, such as the genes responsible for matrix metalloproteinase, tumor necrosis factor alpha, and interleukin 18, whereas the other involves genes implicated in vascular functions, such as angiotensin-converting enzyme gene and vascular growth gene [29]. CAA is a main complication of KD, and many cardiovascular events may be caused by CAA. Therefore, determining the etiology of CAA is important for appropriately diagnosing and treating this disorder.

RAS is one of the most important factors regulating electrolyte balance, and AGT, which is the basis of renin action, is considered a key substance in this system. AGT acts as a substrate to the renin enzyme, which is a part of the renin-angiotensin-aldosterone system (RAAS) in which the N-terminal amino acids of mature AGT secreted by hepatocytes are cleaved intravascularly. Studies have shown that the hyperfunctioning of RAS is closely related to the occurrence of cardiovascular and cerebrovascular diseases [30, 31]. AGT is synthesized by the liver and released into the blood. Under the action of plasma renin, AGT is enzymatically hydrolyzed into angiotensin I (Ang I), which is then degraded into Ang II by ACE. Ang II can stimulate excessive hypertrophy, the proliferation of vascular smooth muscle cells, and increased platelet adhesion, causing vascular injury, such as atherosclerosis and thrombosis. AGT gene polymorphisms have been found to be related to vascular injury-related diseases by researchers in Russia, India, Australia, and China [22, 25, 26, 32]. However, few reports exist on the relationship between AGT gene polymorphisms and the risk of CAA with KD, and the number of cases in these studies is insufficient [7]. In our hospital-based study, we aimed to illuminate this relationship in Southern Chinese children with 760 cases and 972 controls. We chose the most susceptible SNPs after an evaluation of GWAS-identified SNPs related to cardiovascular disease. However, we confirmed that only the rs5050 locus of the AGT gene was associated with susceptibility to CAA in children with KD in this study. The T allele at the rs5050 (T>G) position is the risk locus of the AGT gene (OR > 1). The combined analysis indicated that this genotype increased the CAA risk in children aged < 12 months.

The rs5050 site mutation is located in the promoter region of the AGT gene, which results in a cytosine substituting for adenine (A-20C) at the -20 site. Mutations in polymorphic loci can affect nucleotides and their binding to change the basic transcription rate of the AGT gene. This transcription rate can be increased by 20% to 40% and could result in a significantly higher plasma AGT concentration than that in the no mutation or control group [33, 34]. In this study, we found that the genetic variant rs5050 plays important roles in the development of CAAs during KD progression, although no associations were found between the risk of rs5050 in the AGT gene and KD susceptibility. The genotype increased the CAA risk in KD patients aged < 12 months. To the best of our knowledge, no other studies to date have reported a link between AGT SNPs and the development of CAA in patients with KD, even though SNPs in the AGT gene have been found to be related to cardiovascular disease [7, 35, 36].

We also studied the other mutation, i.e., rs699 (M235T), which is a +704 nucleotide T to C mutation that changes the encoding of the 235th residue; this change leads to the replacement of methionine (M) with threonine (T). The incidence of hypertension and coronary heart disease increased after the nucleotide mutation (T to C) at rs699 [25, 37]. However, the results of our study showed no statistically significant associations between rs699 and the susceptibility to KD compared with that in the healthy controls and no significant associations between rs699 and CAA in children with KD.

In summary, we found that rs699 and rs5050 of the AGT gene were not associated with susceptibility to KD, but rs5050 of the AGT gene was related to CAA risk in children with KD, especially those younger than 12 months. This finding is consistent with the results of some studies showing that KD patients aged ≤ 1 year had a high risk of CAA [35]. The results of this study also showed that AGT gene polymorphisms are among the important factors affecting CAA formation in children with KD. Therefore, based on an analysis of rs5050 in the AGT gene, children with KD may have a predisposition to develop CAA complications and should receive increased attention and early prevention measures. However, the sample size in this study was insufficient because it is difficult to recruit patients with such a rare disease. In addition, this study analyzed patients with KD without formally assessing the population structure of the sampled population. However, neither the relationship between other loci of the AGT gene and CAA nor the associations between rs5050 of the AGT gene and the size of CAA in patients with KD were studied. Therefore, further validation studies are needed to confirm these significant findings.

## Figures and Tables

**Table 1:** Frequency distribution of selected variables among the cases and controls.

Variables	Cases (*n* = 760)		Controls (*n* = 972)		*P* ^a^
	Number	%	Number	%	
Age range, month	1.00-166.00		1.00-166.00		0.133
Mean ± SD	28.04 ± 23.96		26.25 ± 20.84		
<12	231	30.39	326	33.54	
12-60	479	63.03	600	61.73	
>60	50	6.58	46	4.73	
Sex					0.546
Female	252	33.16	309	31.79	
Male	508	68.84	663	68.21	
Coronary artery outcomes					
CAA	104	13.70			
NCAA	656	86.30			

CAA: coronary artery aneurysm; NCAA; no coronary artery aneurysm. ^a^Two-sided *χ*^2^ test of the distributions between the cases and controls.

**Table 2:** Genotype distributions of the AGT gene polymorphism and Kawasaki disease susceptibility.

Genotype/allele	Cases (*N* = 760)	Controls (*N* = 972)	*P* ^a^	Crude OR (95% CI)	*P*	Adjusted OR (95% CI)^b^	*P* ^b^
rs699 (HWE = 0.86)							
AA	15 (1.97)	21 (2.16)		1.00		1.00	
AG	200 (26.32)	269 (27.67)		1.04 (0.52-2.07)	0.91	1.05 (0.52-2.08)	0.899
GG	545 (71.71)	682 (70.16)		1.12 (0.57-2.19)	0.745	1.13 (0.58-2.22)	0.719
Additive			0.776	1.07 (0.89-1.29)	0.418	1.08 (0.89-1.30)	0.439
Dominant	745 (98.03)	951 (97.84)	0.786	1.10 (0.56-2.14)	0.788	1.12 (0.57-2.16)	0.767
Recessive	215 (28.29)	290 (29.84)	0.482	1.08 (0.89-1.33)	0.438	1.09 (0.88-1.34)	0.44
A	230 (15.13)	311 (16.00)	0.486				
G	1290 (84.87)	1633 (84.00)					
rs5050 (HWE = 0.18)							
TT	537 (70.66)	681 (70.06)		1.00		1.00	
TG	203 (26.71)	263 (27.06)		0.98 (0.79-1.12)	0.846	0.98 (0.79-1.21)	0.835
GG	20 (2.63)	28 (2.88)		0.91 (0.51-1.62)	0.742	0.91 (0.51-1.64)	0.763
Additive			0.934	0.97 (0.81-1.16)	0.739	0.97 (0.51-1.64)	0.743
Dominant	223 (29.34)	291 (29.94)	0.786	0.97 (0.79-1.20)	0.789	0.97 (0.79-1.20)	0.784
Recessive	740 (97.37)	944 (97.12)	0.754	0.91 (0.51-1.63)	0.756	0.92 (0.51-1.65)	0.778
T	1277 (84.01)	1625 (83.59)	0.738				
G	243 (15.99)	319 (16.41)					

^a^ *χ* ^2^ test of the genotype distributions between the Kawasaki disease patients and controls. ^b^Adjusted for age and sex.

**Table 3:** Genotype distributions of the AGT gene polymorphism and coronary artery aneurysm among Kawasaki disease patients.

Genotype/allele	CAA (*N* = 104)	NCAA (*N* = 656)	*P* ^a^	Crude OR (95% CI)	*P*	Adjusted OR (95% CI)^b^	*P* ^b^
rs699(A>G)							
AA	1 (0.96)	14 (2.13)		1.00		1.00	
AG	23 (22.12)	177 (26.98)		1.82 (0.23-14.48)	0.572	2.00 (0.25-16.88)	0.515
GG	80 (76.92)	465 (70.88)		2.41 (0.31-18.57)	0.399	2.85 (0.37-20.98)	0.344
Additive			0.365	1.37 (0.87-2.14)	0.174	1.40 (0.89-2.20)	0.142
Dominant	103 (99.04)	642 (97.87)	0.382	2.25 (0.29-17.26)	0.437	2.50 (0.32-19.40)	0.381
Recessive	24 (23.08)	191 (29.12)	0.196	1.37 (0.84-2.23)	0.206	1.40 (0.86-2.29)	0.175
A	25 (12.02)	205 (15.62)	0.178				
G	183 (87.98)	1107 (84.38)					
rs5050(T>G)							
TT	64 (61.54)	471 (71.80)		1.00		1.00	
TG	35 (33.65)	170 (25.91)		1.15 (0.96-2.37)	0.069	1.48 (0.94-2.32)	0.089
GG	5 (4.81)	15 (2.29)		2.45 (0.86-6.98)	0.092	2.58 (0.90-7.39)	0.077
Additive			0.079	**1.53 (1.07**-**2.21)**	**0.021**	**1.53 (1.06**-**2.21)**	**0.024**
Dominant	40 (38.46)	185 (28.20)	0.037	**1.59 (1.04**-**2.45)**	**0.034**	**1.56 (1.01**-**2.41)**	**0.043**
Recessive	99 (95.19)	641 (97.71)	0.172	2.16 (0.77-6.07)	0.147	2.30 (0.81-6.50)	0.117
T	163 (73.37)	1112 (84.76)	**0.020**				
G	45 (21.63)	200 (15.24)					

CAA: coronary artery aneurysm; NCAA: no coronary artery aneurysm. ^a^*χ*^2^ test of the genotype distributions between coronary artery aneurysm and no coronary artery aneurysm among patients with Kawasaki disease. ^b^Adjusted for age and sex.

**Table 4:** Stratification analysis of the association between the rs5050T>G polymorphism and coronary artery aneurysm among patients with Kawasaki disease.

Variables	TT	TG/GG	Crude OR (95% CI)	*P*	Adjusted OR^a^ (95% CI)	*P* ^a^
	CAA/NCAA					
Age, month						
≤12	24/131	19/51	2.03 (1.03-4.03)	0.042	1.94 (0.98-3.87)	0.059
>12	40/340	21/134	1.33 (0.76-2.34)	0.32	1.32 (0.75-2.32)	0.343
Sex						
Female	20/153	14/59	1.82 (0.86-3.83)	0.117	1.85 (0.87-3.90)	0.109
Male	44/318	26/126	1.49 (0.89-2.52)	0.14	1.42 (0.83-2.41)	0.197
Coronary artery outcomes						
GCAA	14/185	21/471	1.70 (0.81-3.84)	0.137	1.35 (0.81-3.36)	0.167
MCAA	15/185	18/471	**2.12 (1.05**-**4.30)**	**0.037**	1.91 (0.924-3.95)	0.080
SCAA	11/185	24/471	1.17 (0.56-2.43)	0.68	1.01 (0.48-2.18)	0.961

GCAA: giant coronary artery aneurysm; MCAA: medium coronary artery aneurysm; SCAA: small coronary artery aneurysm. ^a^Adjusted for age and sex.

## Data Availability

Not applicable. The conclusions of the manuscript are based on relevant datasets, which are available in the manuscript.
